# Breakfast Consumption and Its Association with Insulin Resistance in a Large-Scale Cohort of Children and Adolescents with Overweight/Obesity in Greece

**DOI:** 10.3390/nu17213457

**Published:** 2025-11-01

**Authors:** Odysseas Androutsos, Maria Manou, Ioanna Panagiota Kalafati, Michail Kipouros, Alexandra Georgiou, Evangelia Charmandari

**Affiliations:** 1 Laboratory of Clinical Nutrition and Dietetics, Department of Nutrition and Dietetics, University of Thessaly, 42132 Trikala, Greece; oandroutsos@uth.gr (O.A.); ikalafati@uth.gr (I.P.K.); mihalis.kip@gmail.com (M.K.); 2 Division of Endocrinology, Metabolism and Diabetes, First Department of Pediatrics, “Aghia Sophia” Children’s Hospital, National and Kapodistrian University of Athens Medical School, 11527 Athens, Greece; mariamanou93@hotmail.com (M.M.); alexgeo@hua.gr (A.G.); 3 Division of Endocrinology and Metabolism, Center of Clinical, Experimental Surgery and Translational Research, Biomedical Research Foundation of the Academy of Athens, 11527 Athens, Greece

**Keywords:** breakfast, obesity, insulin resistance, children

## Abstract

**Introduction:** Breakfast skipping has been proposed as a determinant of childhood obesity and disorders of glucose metabolism. The present study aimed to examine the association between breakfast skipping and insulin resistance in children and adolescents with overweight or obesity. **Methods:** In total, 1291 children aged 2–18 years old (boys = 48.4%, boys with obesity = 69.8%; girls = 51.6%, girls with obesity = 60.8%) participated in the study, providing sociodemographic, anthropometric, lifestyle, biochemical, and clinical data. Breakfast consumption was assessed using a validated questionnaire. The IOTF criteria were used to categorize children’s body mass index (BMI) status, while the homeostasis model assessment (HOMA-IR) was used to assess insulin resistance. **Results:** According to the findings of this study, 37.3% of the children/adolescents were found to skip daily breakfast consumption. Girls tended to skip breakfast more than boys (40.5% vs. 33.9%, *p* = 0.016), with the percentage of girls skipping breakfast increasing in the older age groups (2–5 yrs: 27% vs. 6–12 yrs: 39.1% vs. 13–18 yrs: 53.5%, *p* = 0.001). Linear regression analyses showed that breakfast skipping is associated with HOMA-IR in the total sample and in children and adolescents with obesity, after adjusting for several confounding factors (age, gender, Tanner stage, residency, and sports participation). **Conclusions:** A large number of children and adolescents with overweight or obesity, especially adolescent girls, skip daily breakfast consumption, which was associated with insulin resistance. These findings underscore the importance of promoting regular breakfast consumption as a preventive strategy against metabolic complications in children and adolescents with overweight or obesity.

## 1. Introduction

The global prevalence of overweight and obesity in children and adolescents has been displaying a significant rise since the 1980s. According to the latest data from the World Health Organization (WHO), more than 390 million children and adolescents between the ages of 5 and 19 years were classified as overweight, with 160 million of them living with obesity [1]. These trends led to concerns about the long-term public health burden, with childhood obesity being a major predictor of non-communicable diseases in adulthood, including diabetes mellitus type 2, cardiovascular disease, and certain types of cancer [2,3,4,5]. Despite the increasing recognition of the problem, the prevalence of childhood obesity remains at high levels globally, and it is projected to continue contributing to the global burden of disease in the years to come.

One of the factors contributing to the development of childhood obesity is breakfast skipping, a behavior that has been consistently associated with poor dietary patterns and higher caloric intake in the next meals of the day [6]. Skipping breakfast has been linked to an imbalance in energy intake and poor macronutrient distribution, which may lead to overeating later in the day and the consumption of high-caloric/low-nutrient foods [7]. Moreover, skipping breakfast is associated with irregular eating habits, which can disrupt metabolic regulation and increase the risk of developing metabolic disorders, such as insulin resistance [8].

Recent evidence underscores the critical role of breakfast consumption in children’s health. Previous studies indicated that children and adolescents who consume breakfast regularly tend to have better diet quality and a more balanced nutritional profile compared to those who skip this important meal [7]. Those who consume breakfast daily have lower caloric intake, healthier body weight, and better health markers, including glycemic control and lower levels of body fat [7]. This is particularly important given the rising trends of metabolic syndrome and type 2 diabetes among children and adolescents [9]. The study by Gwin et al. highlighted that breakfast consumption is associated with reduced appetite and lower caloric intake throughout the day, thus playing a key role in regulating energy balance [10,11]. As a result, promoting healthy breakfast habits has become a key strategy for the prevention of obesity and non-communicable diseases, emphasizing the need for public health initiatives aiming to improve breakfast consumption among children and adolescents.

Currently, there is limited evidence regarding the potential adverse effects of breakfast skipping on glucose metabolism in individuals living with obesity. We hypothesized that skipping breakfast at least once per week is associated with insulin resistance. The aim of our study was to explore breakfast habits and the association between breakfast skipping and insulin resistance in a large cohort of children and adolescents with overweight or obesity in Greece.

## 2. Materials and Methods

### 2.1. Study Design and Participants

The methodology of the present study has been comprehensively described elsewhere [12]. In summary, the study was conducted between October 2014 and March 2017, enrolling pediatric participants aged 2–18 years with a diagnosis of overweight or obesity, who attended the ‘Center for the Prevention and Management of Overweight and Obesity in Childhood and Adolescence’ at Aghia Sophia Children’s Hospital, Athens, Greece.

Written informed consent was obtained from all participants or their legal guardians prior to inclusion in the study. The research protocol conformed to the ethical principles outlined in the Declaration of Helsinki and the Council of Europe’s Convention on Human Rights and Biomedicine. Ethical approval was granted by the Institutional Review Board of ‘Aghia Sophia’ Children’s Hospital (Approval Number: EB-PASCH-MoM: 28 November 2013, Re: 10290-14 May 2013).

### 2.2. Procedure

Dietary, medical history and socio-demographic data of the participants were collected by trained researchers through structured interviews conducted with parents at the ‘Center for the Prevention and Management of Overweight and Obesity in Childhood and Adolescence’, while blood samples were drawn and analyzed by the hospital health professionals following the standard procedures. Anthropometric measurements, including body weight and height, as well as clinical data (pubertal stage) were obtained by trained pediatricians, using standardized methods and equipment. Children and adolescents diagnosed with diabetes mellitus were excluded from the present study.

### 2.3. Instruments and Variables

#### 2.3.1. Socio-Demographic Data

Data regarding children’s date of birth, gender, and region of residence were provided by their parents. Participants were categorized in different age groups, namely 2–5 years old, 6–12 years old or 13–18 years old. The region of residence was categorized as either “urban” or “rural” based on the family’s reported location.

#### 2.3.2. Anthropometric, Biochemical and Clinical Data

The body mass index (BMI) status of the children was classified according to the International Obesity Task Force (IOTF) criteria [13]. Children with a BMI status classified as “normal BMI” were excluded from the analyses.

Data on fasting blood glucose (mmol/L) and insulin (μUI/mL) concentrations were collected and the homeostasis-model assessment (HOMA-IR) was calculated according to the following equation [14].

HOMA-IR = [IF (μ units/mL^−1^) × GF (mmol/L^−1^)]/22.5

(IF = fasting serum insulin and GF = fasting plasma glucose)

The threshold of 3.16 for HOMA-IR was used to categorize children and adolescents with insulin resistance [15].

The pubertal stage of children was classified according to the Tanner stages [16].

#### 2.3.3. Lifestyle Data

Parents provided information on their child’s food and beverage consumption over the previous 12 months using a food frequency questionnaire (FFQ) that had been validated for the Greek population [17]. The FFQ captured information on the frequency of children’s main meals (breakfast, lunch, dinner) consumption. Parents were asked to report how often their child consumed breakfast, by selecting one of the following categories: “(almost) never,” “1–3 times a month,” “1 day a week,” “2–4 days a week,” “5–6 days a week,” and “every day.” Based on their responses, children were classified as either “daily breakfast eaters” (if they consumed breakfast every morning throughout the week) or “breakfast skippers” (if they did not consume breakfast daily).

Additionally, parents provided information on other lifestyle behaviors of their children through a questionnaire, including the frequency of sports participation, with responses categorized as “Never”, “once/week,” “twice/week,” “three times/week,” or “>three times/week.”

### 2.4. Statistical Analyses

A priori power analysis using G*Power (Version 3.1) for a multiple linear regression model indicated that to detect a small-to-moderate effect size (*f*^2^ = 0.03) of the main predictor at α = 0.05 with 90% power a minimum total sample of 354 participants would be Continuous variables are presented as means with standard deviations (SD), while categorical variables are expressed as relative frequencies (percentages). The normality of the distribution of the variables was assessed through graphical methods (histograms, PP-plots, QQ-plots) and statistical tests (Shapiro–Wilk test). Fasting insulin and HOMA-IR data were non-normally distributed and In-transformed. Linear regression analyses were performed to examine the association between breakfast consumption and insulin resistance (HOMA-IR). All statistical analyses were conducted using SPSS software, version 29.0 (IBM Corp., Armonk, NY, USA), with a significance level set at *p* < 0.05.

## 3. Results

The study sample consisted of 1291 participants (51.6% girls). Table 1 presents the demographic, behavioral and clinical characteristics of the study sample. More specifically, the study sample included children and adolescents from different age groups, ranging from 2 to 18 years old, with the majority of them being 6–12 years old (73.1%). In terms of residency, 12.2% of the participating families lived in rural areas, while the majority (87.8%) were living in urban settings.

Regarding children’s lifestyle, 37.3% of them was found to skip daily breakfast consumption, with a significantly higher number of girls compared to boys adopting this dietary behavior (40.5% vs. 33.9%, *p* = 0.016), and 23.5% of them did not participate in sports. The participants’ pubertal development was assessed using the Tanner stages, with the majority being in the later stages of puberty, i.e., 12.2% in stage V and 7.3% in stage IV. Also, the study sample included a significantly higher number of boys with obesity compared to girls (69.8% vs. 60%, *p* = 0.001).

**Figure 1:**
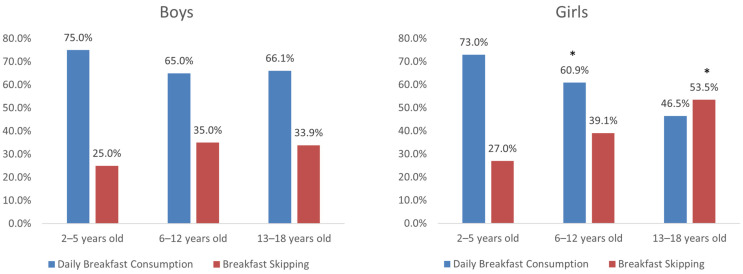
Breakfast consumption/skipping by age group and gender. * *p* < 0.001 for differences in breakfast consumption/skipping between the age categories, in girls.

Figure 1 presents the distribution of children and adolescents consuming or skipping breakfast, according to their gender and age category. The number of children and adolescents skipping breakfast tended to increase with age, with the results indicating a statistical significance only for girls (2–5 yrs: 27.0% vs. 6–12 yrs: 39.1% vs. 13–18 yrs: 53.5%, *p* = 0.001).

The results of the linear regression analyses conducted in this study are presented in Table 2. More specifically, it was observed that breakfast skipping was associated with insulin resistance (HOMA-IR) in the total sample (*p* < 0.05) after adjusting for several potential confounders (children’s/adolescents’ age, gender, Tanner stage, residency and sports participation). After exploring these associations according to children’s/adolescents’ BMI status, the results remained significant only for children/adolescents with obesity.

## 4. Discussion

The present study explored the frequency and association of breakfast skipping with insulin resistance in a large cohort (n = 1291) of children and adolescents with overweight or obesity, in Greece. According to the findings, a large number of children and adolescents, especially adolescent girls, skip daily breakfast consumption. Furthermore, the results of linear regression analyses showed that breakfast skipping is associated with insulin resistance, even after adjusting for several confounders (age, gender, Tanner stage, residency, sports participation).

In line with the findings of the present study, previous studies focusing on the general pediatric population have shown that the number of children or adolescents skipping breakfast is high. More specifically, the ENERGY (EuropeaN Energy balance Research to prevent excessive weight Gain among Youth) project, which included data from 5444 European children 10–12 years old, showed that 12.7% of European children skip their breakfast on weekends and 25.6% on weekdays, while in Greece the relevant percentages were found to be 21.3% and 36.2%, respectively [18]. In adolescence, the number of children skipping breakfast seems to further increase. Moreover, the gender differences observed in our study confirm the findings by Wang et al., which indicated that girls are more likely to skip breakfast than boys [19]. Some possible interpretations for the differences between children include individual factors, such as lack of time, decreased hunger during the morning hours, socioeconomic barriers, health/nutrition literacy and others. In adolescence, issues related to body image/body dissatisfaction are more frequently observed, especially among girls, who tend to adopt unhealthy dieting behaviors as a mean to regulate their body weight, such as eliminating main meals from their diets to decrease energy intake [20,21]. Cultural factors may also influence these patterns, since the societal emphasis on thinness as an ideal of beauty for girls may contribute to variations in their eating behavior and dietary patterns [21].

Breakfast skipping has a significant impact on children’s and adolescents’ health. A recent systematic review of longitudinal studies and randomized controlled trials explored the associations between meal patterns and risk for overweight/obesity in children and adolescents living in Western countries and revealed that regular breakfast consumption may be protective against childhood overweight/obesity, whereas breakfast skipping may negatively influence body weight status [22]. Findings from longitudinal studies [23,24], as well as a review of 16 cross-sectional studies and a recent meta-analysis [25], indicated that breakfast skipping is associated with overweight and obesity in both boys and girls. Another study, which was based on a large sample of children aged 5.6–7.4 years in Iceland, indicated that breakfast skipping was independently associated with overweight/obesity in girls, but not in boys [26]. Interestingly, a prospective study which included a sample of 6529 European schoolchildren, showed that regular breakfast consumption may play a protective role in children’s BMI status [27].

Furthermore, the results of the current study align with the broader body of evidence linking breakfast skipping with insulin resistance and cardiometabolic risk factors. Recent studies revealed an association between regular breakfast consumption and better glycemic control. More specifically, Marlatt et al. reported that breakfast consumption was associated with lower concentrations of fasting insulin and HOMA-IR in a sample of 367 adolescents aged 11–18 years [28]. Similarly, in the study by Jeans et al., frequent breakfast consumption was associated with improved glucose control in children and adolescents [29]. A longitudinal study involving a national sample of Australian children aged 9–15 years examined the associations between breakfast consumption and cardiometabolic outcomes over a 20-year period. The study showed that individuals who skipped breakfast both at baseline and 20 years later had higher concentrations of fasting insulin and HOMA-IR [30]. In Greece, an association between breakfast skipping and insulin resistance was recorded in children 9–13 years old with normal weight, overweight or obesity after adjusting for children’s age and Tanner stage; still this association lost its significance after adjusting for additional confounders [31]. The HELENA-study showed that regular breakfast consumption was associated with healthier cardiometabolic profile (including markers such as HOMA-IR) in European adolescents [32]. Interestingly, the study by Arenaza et al. (2018) showed that higher breakfast energy density from beverages was associated with higher levels of insulin resistance, independently of physical activity levels in children with overweight/obesity [33].

The association between breakfast consumption and obesity is complex, and a clear mechanism has not been established yet. However, regular breakfast consumption may enhance metabolic function, with a fiber-rich breakfast improving postprandial glycemic response, satiety, and insulin sensitivity. Breakfast skipping, on the other hand, may lead to increased total energy intake. A study on adolescents aged 11–16 years found that higher energy intake at breakfast resulted in a decreased overall daily energy intake, while breakfast skipping led to an increased intake of 171 kcal/day. Additionally, children who consume breakfast regularly tend to be more active, which may partially explain the link between breakfast skipping and higher risk for overweight/obesity [34]. Recent reviews also highlight the fact breakfast skipping may promote circadian desynchrony and metabolic dysregulation [35]. Future studies are required to investigate the role of breakfast consumption and chrononutrition in children’s metabolic health.

Considering the important, negative effects of breakfast skipping on children’s nutrient intake, diet quality, metabolism, health, including mental health, and academic performance, it is essential to develop a roadmap for the promotion of breakfast consumption in youth [36,37,38]. Multilevel strategies targeting children’s social (parents/caregivers, teachers, peers) and physical (e.g., home, school, restaurants/cafeterias/markets/areas where children can be served breakfast) environment need to be applied. First, activities to increase health/nutrition literacy of families and awareness of the importance of breakfast consumption for children’s health, especially in vulnerable and low-socioeconomic status families, need to be implemented both by the educational and the health sectors. For very low socioeconomic status (SES) families (e.g., those living in poverty) additional financial actions need to be taken/continued, focusing on the provision of healthy breakfast. It is also important to develop age- and gender- tailor-made strategies, which should be primarily focused -as shown by the present study and previous studies- to adolescent girls. Such strategies should include multidisciplinary approaches, including nutrition education programs and nutrition counseling, which could be applied at community-level with the training and involvement of teachers, pediatricians, dietitians-nutritionists, psychologists/psychiatrists and other health care professionals, as well as the implementation of media campaigns to increase awareness on eating disorders and healthy dietary patterns. Previous studies which used the aforementioned approaches have been found to be effective in increasing breakfast consumption in children and adolescents [39,40,41,42].

The findings of this study should be considered alongside its strengths and limitations. It is one of the few studies to specifically examine the association between breakfast consumption and insulin resistance in a large cohort of children and adolescents with overweight or obesity. Moreover, the use of standardized protocols, methods and tools, as well as the involvement of trained researchers/clinicians in data collection limits the possibility of methodological errors. However, its cross-sectional design prevents the establishment of causal relationships. Additionally, the reliance on self-reported data, such as lifestyle habits, introduces the potential for recall bias or socially desirable responses. The inclusion of additional socioeconomic and lifestyle data, such as sleep duration/quality, was not feasible in this study, although they may affect the relationship of insulin resistance with breakfast habits. It is also noted that data were collected before the COVID-era. Future studies which collected data during and after the COVID-19 period are warranted to confirm the findings of the present study. Furthermore, clinical trials are warranted to elucidate the effects of breakfast consumption on glucose metabolism.

## 5. Conclusions

The present study revealed that a large number of children and adolescents with overweight or obesity skips daily breakfast consumption, and that breakfast skipping is associated with insulin resistance. These findings emphasize the significant role of daily breakfast consumption in maintaining metabolic health and enhancing long-term health outcomes in pediatric populations. Moreover, girls were more likely to skip daily breakfast consumption compared to boys, especially in the age groups of 6–12 and 13–18 years, highlighting the need for gender- and age- specific strategies to promote healthier dietary habits.

## Figures and Tables

**Table 1:** Demographic, behavioral, and clinical characteristics of the study sample.

	Total (n = 1291)	Boys (n = 625)	Girls (n = 666)	*p*-Value
Age (%)				0.015
2–5 years	7.8	5.4	1.2	
6–12 years	73.1	75.7	70.5	
13–18 years	19.1	18.9	19.3	
Residence (%)				0.221
Urban	87.8	88.9	86.7	
Rural	12.2	11.1	13.3	
Breakfast skipping (% Yes)	37.3	33.9	40.5	**0.016**
Sports participation (%)				**4 × 10^−6^**
Never	23.5	18.0	29.5	
Once/week	4.3	4.4	4.1	
Twice/week	19.7	17.3	22.4	
Three times/week	30.7	33.2	27.9	
>Three times/week	21.8	27.1	16.1	
Tanner stage (%)				**1.023 × 10^−15^**
I	52.7	60.0	45.7	
II	18.5	19.9	16.9	
III	9.3	9.4	9.5	
IV	7.3	6.4	7.9	
V	12.2	4.3	20.0	
Overweight (%)	34.9	30.2	39.2	**0.001**
Obesity (%)	65.1	69.8	60.8	
Fasting glucose (mmol/L)	4.5 ± 0.4	4.5 ± 0.4	4.4 ± 0.4	**2.5 × 10^−5^**
lnFasting insulin	2.6 ± 0.7	2.5 ± 0.6	2.6 ± 0.7	**0.043**
lnHOMA-IR	0.95 ± 0.68	0.92 ± 0.66	0.98 ± 0.69	0.163

Statistically significant differences are highlighted in bold.

**Table 2:** Linear regression analyses of the association between breakfast skipping and HOMA-IR values in children and adolescents with overweight and obesity.

	Total				Overweight			Obesity	
	Beta	SE	*p*	Beta	SE	*p*	Beta	SE	*p*
Breakfast skipping	0.092	0.046	0.045	0.004	0.075	0.957	0.109	0.055	0.047

All models have been adjusted for age, sex, Tanner stage, residency and sports participation (frequency/week).

## Data Availability

The data presented in this study are available on request from the corresponding author. The data are not publicly available due to ethical restrictions.
